# HATS5m as an Example of GETAWAY Molecular Descriptor in Assessing the Similarity/Diversity of the Structural Features of 4-Thiazolidinone

**DOI:** 10.3390/ijms23126576

**Published:** 2022-06-12

**Authors:** Mariusz Zapadka, Przemysław Dekowski, Bogumiła Kupcewicz

**Affiliations:** 1 Department of Inorganic and Analytical Chemistry, Faculty of Pharmacy, Nicolaus Copernicus University in Toruń, Jurasza 2, 85-089 Bydgoszcz, Poland; 2 New Technologies Department, Softmaks.pl Sp. z o.o., Kraszewskiego 1, 85-241 Bydgoszcz, Poland; przemyslaw.dekowski@softmaks.pl

**Keywords:** QSAR, H-GATEWAY, HATS, 4-thiazolidinones, molecular descriptors, interpretation

## Abstract

Among the various methods for drug design, the approach using molecular descriptors for quantitative structure–activity relationships (QSAR) bears promise for the prediction of innovative molecular structures with bespoke pharmacological activity. Despite the growing number of successful potential applications, the QSAR models often remain hard to interpret. The difficulty arises from the use of advanced chemometric or machine learning methods on the one hand, and the complexity of molecular descriptors on the other hand. Thus, there is a need to interpret molecular descriptors for identifying the features of molecules crucial for desirable activity. For example, the development of structure–activity modeling of different molecule endpoints confirmed the usefulness of H-GETAWAY (H-GEometry, Topology, and Atom-Weights AssemblY) descriptors in molecular sciences. However, compared with other 3D molecular descriptors, H-GETAWAY interpretation is much more complicated. The present study provides insights into the interpretation of the HATS5m descriptor (H-GETAWAY) concerning the molecular structures of the 4-thiazolidinone derivatives with antitrypanosomal activity. According to the published study, an increase in antitrypanosomal activity is associated with both a decrease and an increase in HATS5m (leverage-weighted autocorrelation with lag 5, weighted by atomic masses) values. The substructure-based method explored how the changes in molecular features affect the HATS5m value. Based on this approach, we proposed substituents that translate into low and high HATS5m. The detailed interpretation of H-GETAWAY descriptors requires the consideration of three elements: weighting scheme, leverages, and the Dirac delta function. Particular attention should be paid to the impact of chemical compounds’ size and shape and the leverage values of individual atoms.

## 1. Introduction

QSAR models combine different computational and multivariate statistical or machine learning techniques to establish the relationship between structure and biological activity, physicochemical properties, or toxicity of compounds. The main task of QSAR analysis, namely predicting the activity or properties of new compounds, is accomplished in many applications thanks to dynamically developing computational methods. Recently, comprehensive reviews about the development of QSAR modeling and its role in different fields have been published [1,2,3]. During the last decades, the pharmaceutical industry has been greatly interested in analyzing large chemical databases, and machine learning techniques can accelerate and automate the exploration of a large amount of available data. Thus, computer advances and high-throughput screening technologies in drug discovery revolutionized the computational analysis of bioactive molecules [4,5]. For years, such studies have also focused on how to theoretically capture and transform information encoded in a molecular structure into a numerical value readable by computers and understandable for scientists.

The first reports dedicated entirely to the theory of the GEometry, Topology, and Atom-Weights AssemblY (GETAWAY) descriptors were published in 2002 [6,7]. The presentation of GETAWAY descriptors was an attempt to catch as much chemical and structural information as possible. GETAWAY appeared to be a good alternative to popular traditional topological indices accounting for three-dimensional representation of the molecule by including geometric information (3D-Wiener index, the Randic molecular profiles, BCUT descriptors). Consonni and co-workers [6] proposed a new molecular representation calculated from the spatial coordinates of the molecule atoms in a chosen conformation, allowing for a more accurate similarity/diversity analysis of compounds stored in databases. The development of structure–activity modeling of different biological/physicochemical endpoints confirmed the usefulness of GETAWAY descriptors in molecular sciences [8,9,10,11,12,13,14]. Several studies prove that the combination of GETAWAY and WHIM descriptors significantly improves the predictive power of the QSAR model [15,16,17,18]. However, one crucial issue hampers GETAWAY’s more extensive application in QSAR studies. The GETAWAY values, as such, deliver no detailed information about direct contributions from individual atoms or molecular fragments to the final descriptor value. 

The ability to create a direct link between moieties, molecular descriptors, and biological activity should be an integral part of the research [19,20]. Unfortunately, the H-GETAWAY interpretation usually contains some shortcomings [21,22,23,24,25]. The problem lies in interpreting H-GETAWAY descriptors based mainly on leverages, which are not computed but estimated from a visual assessment of the molecular shape. The leverages of the molecule depend on molecular size and shape; thus, comparing leverages between compounds that differ in the number of atoms can induce misinterpretation. In addition, the atomic pairs indicated by the Dirac-delta function are not investigated for autocorrelated GETAWAY. Therefore, the simultaneous consideration of the weighting scheme, leverages, and the Dirac delta function is required to avoid the above shortcomings. Demonstrating how various structural features translate into descriptor values requires performing additional calculations for the complex analysis of these three components. Thus, there is a systematic need for a tool supporting GETAWAY interpretation. This class of molecular descriptors can be sub-classified based on the matrix type: H-GETAWAY (molecular influence matrix H) and R-GETAWAY (influence/distance matrix R). A good example of a thorough interpretation of the R-GETAWAY third-order autocorrelation index weighted by the atomic mass descriptor (R3m) was recently published [8]. Unfortunately, such a detailed interpretation of descriptors in QSAR models appears relatively rarely in the studies.

This paper presents a comprehensive analysis of the similarity/diversity of the structural features of 4-thiazolidinone derivatives using HATS5m (leverage-weighted autocorrelation with lag 5, weighted by atomic masses) molecular descriptor (belonging to the H-GETAWAY type). The first section briefly summarized the definitions, formulas, and mathematical properties of HATS descriptors. The second part illustrates the interpretation of HATS5m for Random Forest and Gaussian processes regression models of 4-thiazolidinones antitrypanosomal activity presented in [26]. This study adopted a substructure-based approach to explore how the weighting scheme of atoms, size, and shape of molecules affects the HATS5m value. 

## 2. Computational Methods

### 2.1. Description and Calculation of HATS Indices

The calculation algorithm for HATS indices utilizes information about molecular structure in two different ways: topological and geometrical. To fully understand both ways of encoding chemical information through HATS indices, explaining the ideas of the molecular graph, Dirac-delta function, Molecular Influence Matrix, and leverages is necessary. Topological reasoning involves visualizing the molecular structure as a graph, where the vertices correspond to atoms while the edges represent bonds. The molecular graph representation is 2D data with no spatial relationships between atoms. The number of bonds (edges) between atoms (vertices) on the shortest possible path (topological distance) is determined topological distance matrix. The QSAR equation supplies information on what topological distance (*lag*) is relevant to the biological activity under study. The Dirac-delta function selects only those atomic pairs within a distance indicated by the QSAR model. The Dirac-delta function is defined by

(1) $$\delta \left(k;d_{ij}\right)=\{\begin{array}{c} 1\;if\;d_{ij}=k\\ 0\;ifd_{ij}\neq k \end{array}\;,$$

where *d_ij_* is the topological distance between the *i*th and *j*th atoms and *k* = 1, 2, …, *d*. 

The second source of molecular structure information is centered Cartesian coordinates, which efficiently depicts molecular geometry. The leverage matrix is determined based on centered Cartesian coordinates, namely, the Molecular Influence Matrix (MIM).

The *Molecular Influence Matrix* (MIM), denoted by *H*, is defined in terms of the *molecular matrix M* as

(2) $$H=M\cdot {\left(M^{T}\cdot M\right)}^{-1}\cdot M^{T},$$

where the superscript *T* refers to the transposed matrix. The *M* matrix is formed from *A* rows corresponding to the atoms in a molecule and three columns corresponding to the centered Cartesian atomic coordinates *x*, *y*, *z*. The molecules should have explicit hydrogen atoms. GETAWAY descriptors are invariant to translation and rotation due to the calculation of atomic coordinates concerning the geometrical center of the molecule. In the context of molecular structure, the *H* matrix evaluates the impact of atoms on each other. The diagonal elements of the symmetric *H* matrix, *h_ii_*, are called leverages and satisfy:

(3) $$0\leq h_{ii}\leq 1$$

(4) $$\overset{A}{\underset{i=1}{\sum }}h_{ii}=D$$

(5) $$\bar{h}=\frac{D}{A}$$

where *h* denotes elements of the molecular influence matrix, *A* is the number of atoms, *D* is the rank of the molecular matrix, and $\bar{h}$ is an average value of the diagonal terms. 

The 3D-molecular geometry is the mutual distribution of atoms in the space around the molecular center. Leverage expresses how much the *i*th atom impacts the molecular shape of the whole molecule. The non-diagonal elements have a value in the [–1, 1] range. A large value of *h_ii_* (high leverage) indicates that the *i*th atom is distant from the center of the Cartesian coordinates of the molecule. Molecular Influence Matrix (MIM) is a convenient tool for considering information about the size and shape, especially concerning symmetry and molecular branches. In a series of molecules with similar sizes, lower leverages can be found for atoms in molecules of spherical shape than in more linear ones. It can be explained by a significantly smaller total distance of atoms from their geometrical center. In a set of compounds with similar shapes, an increase in the number of atoms cause a decrease in leverages [27,28]. 

The topological and geometrical approaches mentioned above describe only the molecular structure without considering the physicochemical properties of the atoms. This limitation may be removed by using atom weights. For example, the weighting schemes encode atomic mass (m), atomic polarizability (p), atomic electronegativity (e), or van der Waals atomic volume (v). All of them are scaled up by relating the carbon atom, which weight equals one. 

The HATS indices are defined as autocorrelation descriptors and calculated according to the following equations [28]:

(6) $$HATS_{0}\left(w\right)=\overset{A}{\underset{i=1}{\sum }}{\left(w_{i}\cdot h_{ii}\right)}^{2}$$

(7) $$\begin{array}{c} HATS_{k}\left(w\right)=\sum_{i=1}^{A-1}\sum_{j>i}\left(w_{i}\cdot h_{ii}\right)\cdot \left(w_{j}\cdot h_{jj}\right)\cdot \delta \left(k;d_{ij}\right)\\ k=1,2,\ldots ,d \end{array}$$

where *A* is the number of atoms, *w_i_* and *w_j_* are the weights of the *i*th and *j*th atoms, *h* denotes elements of the molecular influence matrix (leverages), δ means Dirac delta, and *d_ij_* is the topological distance between the *i*th and *j*th atoms for respective *lag* (k). Usually, k is from 1 to 8. 

**Figure 1:**
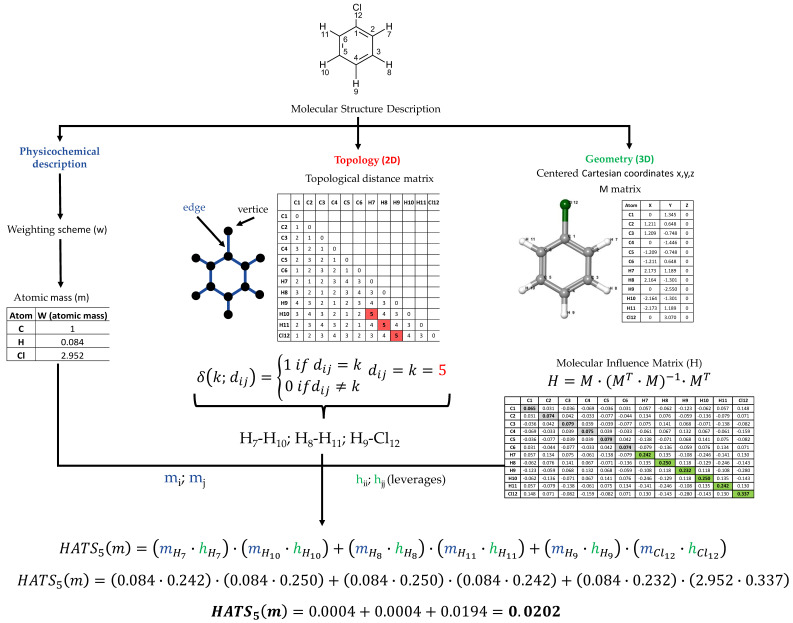
Scheme of HATS5m value calculation for exemplary molecule—chlorobenzene [28].

A graphic illustration of the HATS (i.e., HATS5m) descriptor value calculation for an exemplary molecule (chlorobenzene) is shown in Figure 1. HATS5m means the leverage-weighted autocorrelation descriptor calculated for atoms spaced at five bonds (*lag* 5) and weighted by atomic masses (m). 

### 2.2. The (Mis)Interpretation of HATS Descriptor Based on the Concept of Leverage

Evaluating QSAR model quality is ubiquitous and essential in many disciplines, such as risk assessment, toxicity prediction, drug discovery, and drug optimization [29,30]. However, guidelines for interpreting molecular descriptors, especially autocorrelation 3D descriptors, have not been fully proposed. In most cases, the interpretation of HATS indices is primarily limited to analyzing the relationship between molecular shape and final descriptor value. Additionally, currently available software for calculating molecular descriptors does not provide accurate, explicitly leverage values [31,32,33,34,35]. Instead, it assumes that peripheral atoms in molecules have higher leverage values than atoms closer to the geometric center. Disregarding the role of the Dirac-delta function and molecular size may result in misinterpretation. 

**Figure 2:**
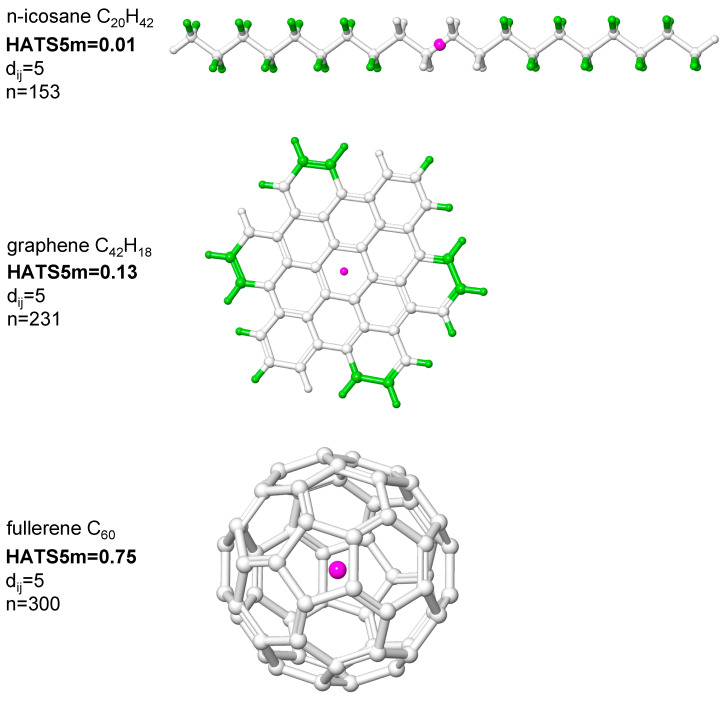
HATS5m values for molecules with various shapes: n-icosane C_20_H_42_, fullerene C_60_, and graphene-like structure C_42_H_18_; *d_ij_*—the topological distance between the *i*th and *j*th atoms; n—number of atomic pairs spaced at *d_ij_* distance. Atoms with the highest value of leverage are depicted in green. The geometric center of the molecule is marked pink.

In this study, we focused on the HATS descriptor calculated for atoms spaced at five bonds (*lag* 5) and weighted by atomic mass, i.e., HATS5m. To illustrate the impact of the Dirac-delta function on the HATS5m value, we considered the exemplary molecules with similar sizes (approximately 60 atoms) and various shapes: n-icosane C_20_H_42_, fullerene C_60_, and graphene-like structure C_42_H_18_ (Figure 2). 

The n-icosane is acyclic, an unbranched hydrocarbon with elongated conformation, while the fullerene C_60_ is a hollow sphere. A single layer of carbon atoms arranged in a honeycomb structure forms the graphene-like sheet. Green highlighting indicates atoms with high leverage scores. In line with the generally accepted approach [6], lower leverages can be found for atoms in fullerene C_60_ (spherical shape) than in linear n-icosane. Hence, it could be expected that fullerene may have the lowest HAT5m value among these compounds, but contrary to this, significantly different values are obtained. In fact, fullerene’s descriptor value is greater (0.75) than n-icosane (0.01). HATS5m was calculated only for atoms spaced at five bonds (as an arbitrary choice) for these compounds. Based on the Dirac-delta function, these compounds are differentiated by the number of atomic pairs distinct at five bonds (*lag* 5): 300, 231, and 153 for fullerene, graphene-like structure, and n-icosane, respectively. Many atoms in a specified distance with relatively low leverages in fullerene translate to a much higher descriptor value than n-icosane and a graphene-like sheet containing a small number of atoms with high leverages at the periphery structure. In addition, the weights of atoms also have a significant impact on the descriptor value of these compounds. The low HATS5m value of graphene-like structure compared to fullerene C_60_ results from high leverage and low weight values of hydrogen atoms. Therefore, the sum of the low weights and high leverages products translates to a low descriptor value (see Equation (7)). The interpretation of the HATS indices and the correlation with shape should combine all elements: weighting scheme, leverages, number, and distribution of atomic pairs separated by a given topological distance (obtained from Dirac delta).

## 3. Results and Discussion

Despite the GETAWAY descriptor’s proven usefulness, a more detailed analysis seems needed, particularly regarding the scope, interpretation, and practical application. To better understand and interpret the HATS5m (leverage-weighted autocorrelation with *lag* 5, weighted by atomic masses), we took the molecular structures and their biological activity from the study of Kryshchyshyn and co-workers [26]. They summarized quantitative structure–activity relationship (QSAR) data on the antitrypanosomal activity of 206 different thiazolidinone derivatives using the four predictive models. Random Forest and Gaussian processes regression built comparable predictive models among four tested machine learning algorithms. The HATS5m was one of the essential molecular descriptors in these models. Its relative importance is 95.66 (in the Gaussian processes model) and 77.29 (in the Random Forest model). According to the results obtained in [26], both low and high values of HATS5m were related to the increase in the antitrypanosomal activity.

### 3.1. The Concept of Substructure-Based Method

**Figure 3:**
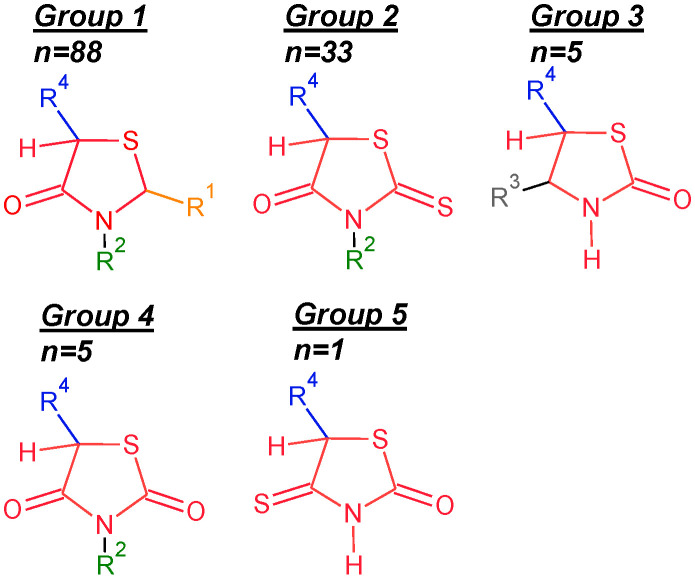
The general structure of five groups of 4-thiazolidinone derivatives with particular fragments marked different colors: common cores (Φ)—red, and substituents R1—orange, R2—green, R3—gray, R4—blue.

The molecular structures of the 4-thiazolidinone derivatives reported in [26] differ significantly regarding their common core (Φ) and substituents. Therefore, we chose a structurally consistent group of 132 compounds from 206 to analyze and improve the interpretability of the HATS5m (Structures-1.sdf in Supplementary Materials). The series of compounds contains the essential substituents, which change logically, systematically, and rationally (Figure 3). Analyzing a smaller and more homogeneous data set (*n* = 132) can help capture and understand the molecular features of compounds encoded in the model constructed in [26].

**Figure 4:**
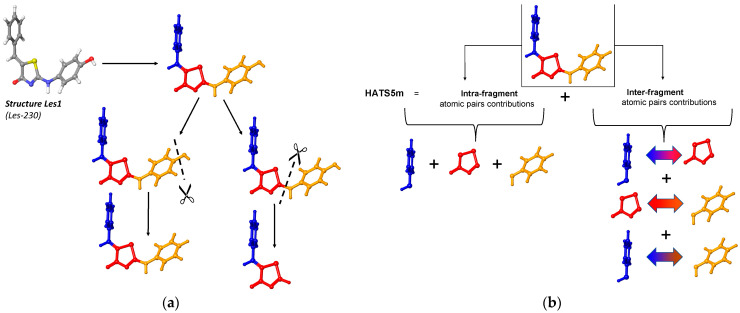
Visual explanation of the substructure-based approach: (**a**), HATS5m as the sum of the individual fragment’s contributions: intra- and inter-fragment effects (**b**).

The commonly used programs such as DRAGON, Mordred, and ChemoPy produce the final value of the descriptor but do not provide any insight into the partial results. Improving the profound understanding of H-GETAWAY change requires additional calculations to get these partial results. Thus, we adopted a **substructure-based** method as a straightforward way to explore how the changes in molecular features of compounds affect the HATS5m value (Figure 4a). 

The substructure-based method works by dividing a molecule into molecular fragments. The value of HATS5m is the sum of the individual fragment’s contributions and the contributions resulting from their mutual spatial arrangement, respectively, intra- and inter-fragment effects (Figure 4b). It is a very convenient way of defining the contribution not only of each fragment, but also to demonstrating the importance of their mutual spatial relationships in the HATS5m value. 

Additionally, applying the leverage concept requires the cutting of molecules (Figure 4a). Leverage describes the degree of participation of all atoms in determining the spatial structure of the compound; thus, the individual values of leverages are strictly assigned to the geometry of the molecule. For example, the same moiety but surrounded by a various number of atoms have a significantly different set of leverages. Therefore, we adopted the substructure-based method combined with the cutting of molecules, which involves modifying the molecular structures of 4-thiazolidinone derivatives reported in [26]. The prefix *Sub* denotes post-cut structures in contrast to the source compounds *Les*. The substructure-based approach allows us to keep a structurally homogeneous set of compounds with the same common core and slight modifications among substituents. In this case, a similar size of obtained compounds provides comparable leverage values.

In the beginning, the 4-thiazolidinone derivatives were characterized according to the selected fragments. Each molecule was divided into five fragments: common core (Φ) 4-thiazolidinone and substituents R1, R2, R3, and R4. The set of compounds was split into five subsets with different common cores (Φs) (Figure 3). To better readability, molecular fragments were highlighted in different colors: Φ—red, R1—orange, R2—green, R3—gray, and R4—blue.

The substructure-based approach emphasizes that the common cores of 4-thiazolidinone derivatives (n = 132) have an unequal impact on the descriptor value. The minimum and maximum HATS5m values are 0.0329 and 0.9343, respectively, while the median value is 0.1063. Table S1a,b summarize the contributions of fragments, including the influence of their spatial arrangement. Fifteen compounds are selected following the highest and lowest descriptor value. The values in Table S1 indicate that all common cores (red) do not impact the HATS5m, resulting from a lack of atomic pairs spaced at a topological distance equal to five in their structures. The exact impact of common core structures depends on their spatial arrangement relative to individual fragments. For both the lowest and the highest values of the descriptor, the most important is the inter-fragment relations between common cores and substituents R2 (green) and R4 (blue) because of interactions of the divalent sulfur atom in Φ with the atoms in R2 and R4. Most contributions originating from the R1 (orange) and R3 (gray) groups do not seem relevant. However, the R1 substituents attached to the C2 atom of the common core of group 1 cause a significant increase in the molecular size and a decrease both in the leverage values and HATS5m. Only in the case of Les-4016, the common core of group 1 is important due to Φ-R1 contribution (Table S1). Common cores (Φ) for groups 3 and 5 are not substantial as these compounds have no R2 (green) substituent. 

In a subsequent part of the article, we focused on assessing the impact of the weights of atoms (Section 3.2), size (Section 3.3), and shape (Section 3.4) of the molecule on the HATS5m value.

### 3.2. The Impact of Atom-Weights (w_i_, w_j_)

Unweighted descriptors treat each atom equally, while weighting is intended to distinguish between the atoms. For calculating the molecular descriptor, atomic weighting schemes involving specific atomic properties are used. The atomic properties often used as atomic weights are: atomic mass (m), van der Waals volume (v), the electronegativity of the atom (e), atomic polarizability (p), and ionization potential (i).

**Figure 5:**
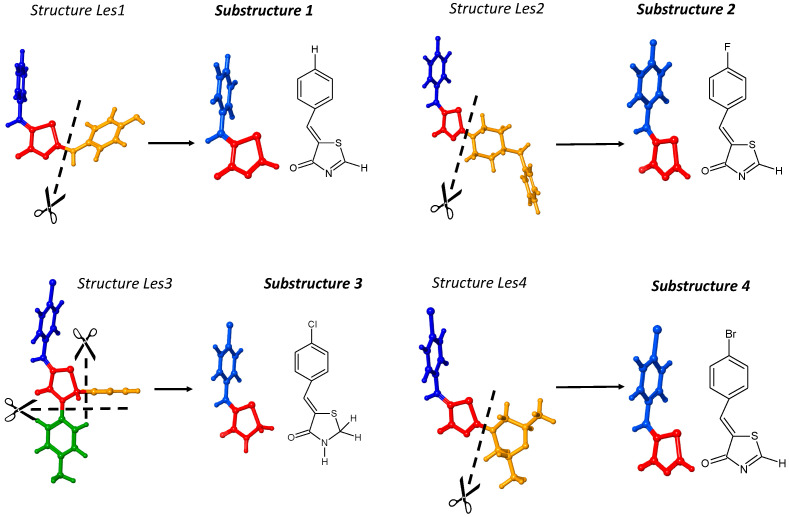
The cutting of the compounds Les1, Les2, Les3, and Les-4 leads to substructures 1, 2, 3, and 4, respectively. Common core is highlighted in red, and substituent R4 is blue.

The effect of atomic mass on the HATS5m value is investigated using the compound Les1 (label in [26] is Les-230) and three halogenated 4-thiazolidinones derivatives: Les2 (Les-3909), Les3 (Les-3201), and (Les4) Les-3975 (Figure 5). After removing R1 (orange) and R2 (green) substituents from these compounds, we obtained four substructures differing only in the substituent in the phenyl ring (except for substructure 3, having two more hydrogen atoms than in other molecules). The leverage value of a halogen atom (and its contribution to the HATS5m value) in starting compounds is affected by the rest of the molecules. Still, the obtained substructures 1–4 ensure that the contribution of the individual atoms, including halogen, is unbiased.

**Figure 6:**
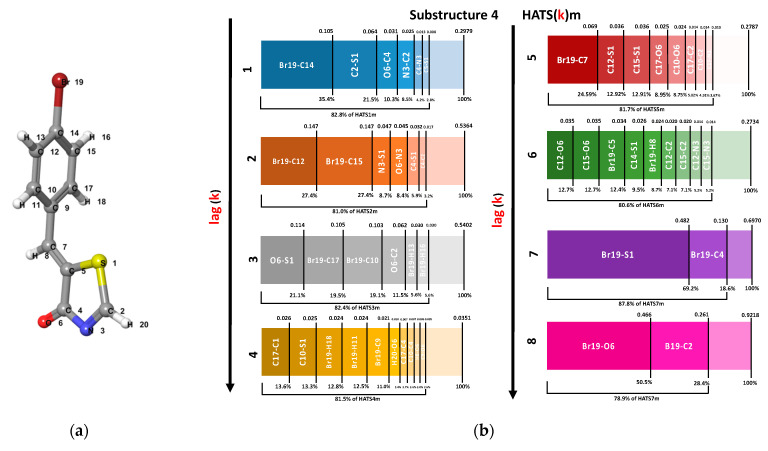

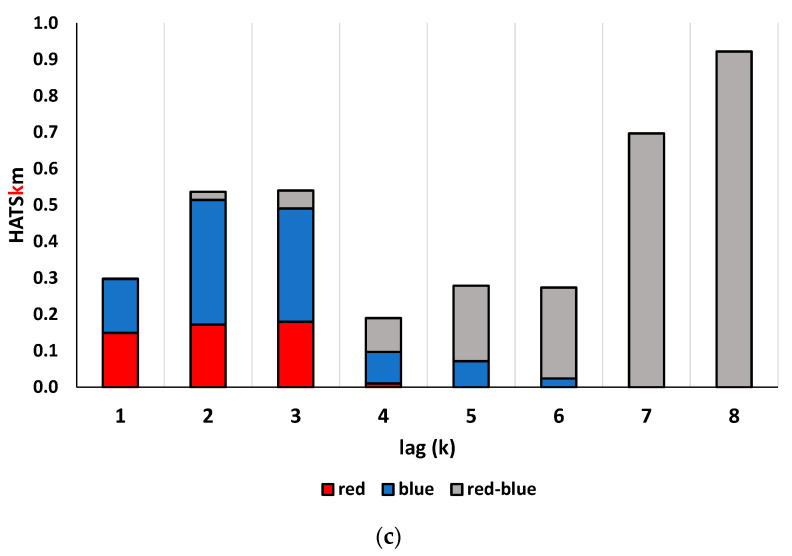
(**a**) Substructure 4 with numbered atoms; (**b**) Impact of lag value (k) on atomic pairs with the largest contribution to the descriptor’s value for substructure 4; (**c**) Contributions from atomic pairs within the R4 (blue) fragment and between the common core and R4 (red-blue), which covered 100% of HATS(k)m value for different lag (k).

The interpretation of the HATS descriptor is based on identifying the atomic pairs with the largest contribution to the descriptor’s value. Figure 6 shows atomic pairs with the largest contribution to the atomic mass-weighted descriptor HATS calculated for different lag values (from 1 to 8). For example, substructure 4 (from compound Les4) has 25 atomic pairs at the topological distance *d_ij_* = 5, and the sum of their contributions gives the final value of the HATS5m descriptor. Only eight atomic pairs account for 80% of the HATS5m value. See the Supplementary Video (Video S1) to overview the most valuable atomic pairs of substructure 4. As shown in Figure 6b, depending on the k value (*d_ij_*), the different number of atomic pairs covers 80% of the descriptor value. In addition, the descriptor values from HATS1m to HATS8m also differ. Figure 6c presents contributions of atomic pairs from different molecular fragments to the HATS(k)m value. 

**Figure 7:**
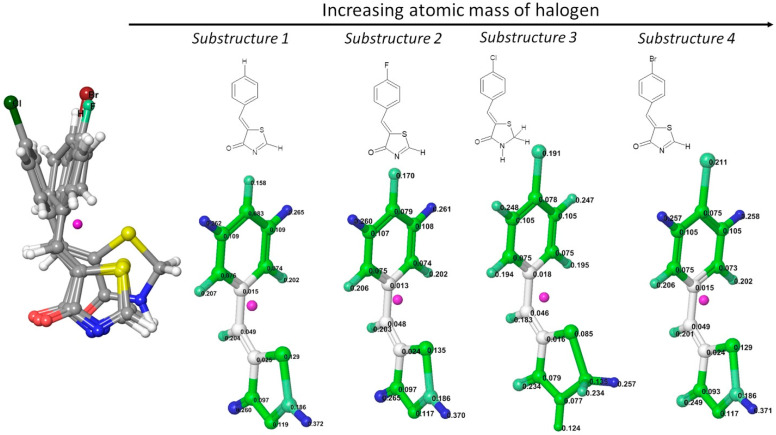
Substructures of compounds Les1, Les2, Les3, and Les4. The geometric center of the molecule is marked pink. Each atom in the molecular structure has a label with a leverage value. Color highlighting indicates atoms with high (blue), medium (green), and low (white) leverage scores.

Figure 7 shows substructures 1–4 with centered cartesian coordinates. All molecules share the exact geometric center (pink dummy atom) and have the same size. The differences in the descriptor values are due to the type of atom numbered 19 (H, F, Cl, or Br), its weight, and the spatial distribution of other atoms. Atoms are highlighted in color according to the value of the leverage. For example, atoms near the geometric center of the molecule are highlighted in white; the most influential atoms with the highest leverages are blue, whereas the intermediate values are green. The leverages pattern in Figure 7 indicates that the hydrogen in the meta position of the benzylidene substituent has a higher leverage value than the halogen in the para position.

**Figure 8:**
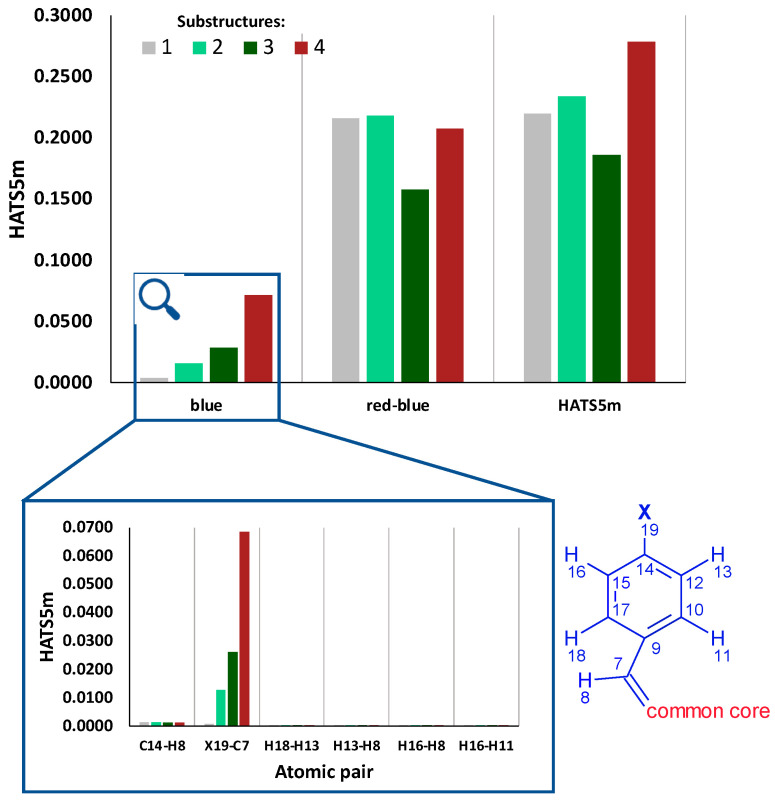
The contribution of intra- and inter-fragments effects in the HATS5m value. In magnification: the impact of atomic pairs in blue (R4) fragment on HATS5m value. The letter “X” represents the halogen atoms F, Cl, and Br in substructures 2, 3, and 4, respectively.

All substructures consist of two fragments: common core (red) and benzylidene substituents (blue). Figure 8 and Table S2 present the contribution of intra- and inter-fragments effects in the HATS5m value. Additionally, Figure S1 shows the contribution of specific red-blue atomic pairs in HATS5m. The descriptor value consists mainly of contributions from red-blue (Φ-R4) atomic pairs, which demonstrate the crucial importance of mutual spatial arrangement of Φ and R4.

The differences in the HATS5m value of substructures 1-4 are mainly related to the blue fragment (R4). Figure 8 (the inner box) shows that the main contribution is that the HATS5m value has only one atomic pair (X19-C7) spaced at the topological distance equal to five. The contribution of the atomic pair H19-C7 (in substructure 1) to the descriptor value is 0.0007. In contrast, the contribution of the atomic pairs F19-C7, Cl19-C7, and Br19-C7 increases with the increase in the halogen atomic mass and equals 0.0128, 0.0261, and 0.0685, respectively. The greater the atomic mass of a halogen, and hence its weight, the more significant its contribution to the descriptor value. Similarly, in the case of the R3m descriptor (R-GETAWAY third-order autocorrelation index weighted by the atomic mass), the presence of atoms heavier than C in a molecule results in a greater contribution to the R3m value [8]. Note, according to Equation (7), each atom’s contribution to HATS5m depends on both the atom weight (atomic mass in this case) and the leverage. The value of the leverage, in turn, depends on the position of the atom in the molecule.

Therefore, we want to check whether introducing a halogen atom in the meta ring position changes the leverage value and the HAST5m descriptor. Geometry optimization is not performed to maintain reproducible conditions. Figure S2 shows the effect of introducing a Br atom into the ring at the meta position on the value of the HAST5m descriptor. The HATS5m meta-isomer value increases by about 21% compared to the para-isomer (detailed information in Supplementary Materials, Figure S3). 

Apart from changing the *lag*, the HATS descriptor value also depends on the type of atomic weightings. To show the weighting scheme impact, we calculated the contributions of atomic pairs (five bonds distant) to HATS5(w) descriptor values, by using the atomic mass, electronegativity, ionization potential, and van der Waals volume. For comparison, the atomic pair contributions to the unweighted HATS5 descriptor value (i.e., HATS5u) were computed. Results are presented in Table S3 (in Supplementary Materials). Weighted descriptors are sensitive to the presence of specific molecular fragments, unlike unweighted. Atomic mass significantly increases the effect of P, S, and Cl and heavy atoms such as Br or I. Similarly, van der Waals volume and polarizability decrease the effect of H atoms and increase contributions of sulfur and bromine (the most influential atomic pairs Br19-C7 and C12-S1, contain Br and S atoms).

**Figure 9:**
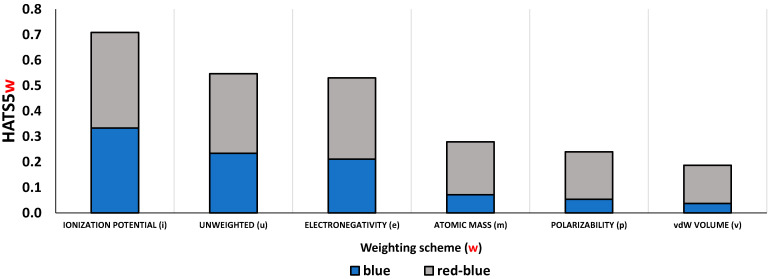
Contributions from atomic pairs within the R4 (blue) fragment and between the common core and R4 (red-blue) to the HATS5 value for different atomic weighting.

In turn, Figure 9 shows how eight atomic pairs that cover 80% of the HATS5m value change their contribution to the descriptor values if the weighting scheme changes. Contributions from atomic pairs within the R4 (blue) fragment and those between the common core and R4 (red-blue) were distinguished.

### 3.3. Impact of Molecular Size

**Figure 10:**
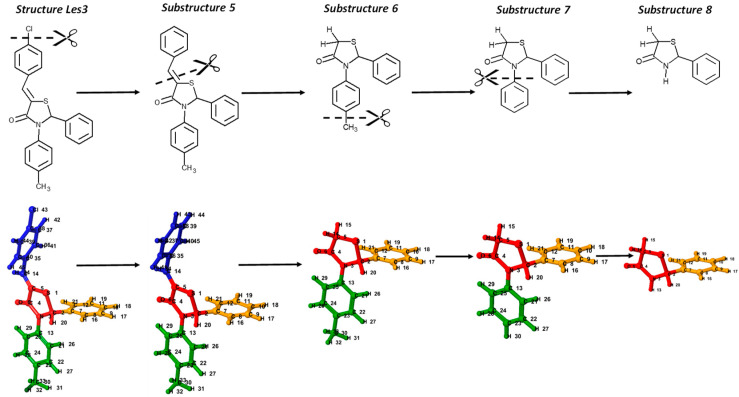
The cutting scheme of the molecule Les3 (Les-3201). The bottom row shows the numbering of atoms and color codes of common core (Φ—red) and substituents (R1—orange, R2—green, and R4—blue).

**Figure 11:**
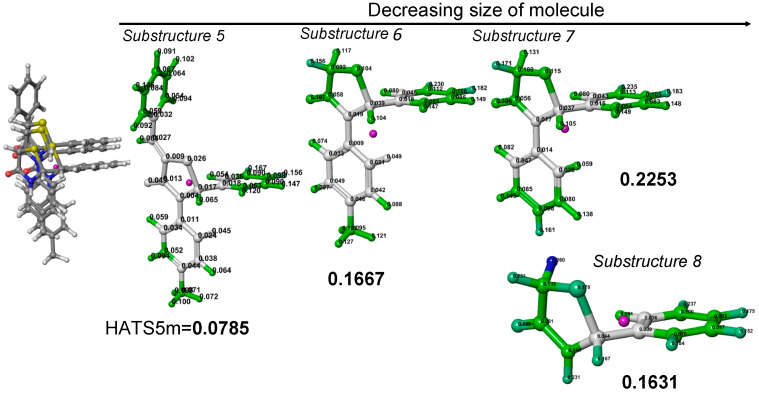
Illustration of the effect of reducing molecule size on increasing leverage scores and changes in HATS5m value. Each atom in the molecular structure has a label with a leverage value. Color highlighting indicates atoms with high (blue), medium (green), and low (white) leverage scores.

The substructure-based analysis of the Les3 examines the effects of molecular size on the HATS5m value. The Les3 is decomposed into moieties treated as new independent structures numbered 5, 6, 7, and 8 (Figure 10). The size of a molecule is a critical element of leverage analysis; generally, as the molecule size decreases, the leverage scores increase (Figure 11). Substructure 5 is formed by cutting off a chlorine atom from the Les3 molecule. Due to the lack of chlorine atom, the HATS5m value of substructure 5 (0.0785) slightly decreases (app. 8%) compared to Les3 (0.0859). Downsizing of molecules, from 5 through 6 to 7 substructures, is accompanied by increasing leverages, and consequently, the HATS5m value also gradually increases. Enhancement of the HATS5m value for six and seven is caused by the rising leverage of atom pairs in R1-Φ (orange-red) and R3-Φ (green-red) fragments. Further reduction in the molecular size (from substructure 7 to 8) translates into a significant decrease in the number of pairs distant by five bonds, which decreases the descriptor value. 

**Figure 12:**
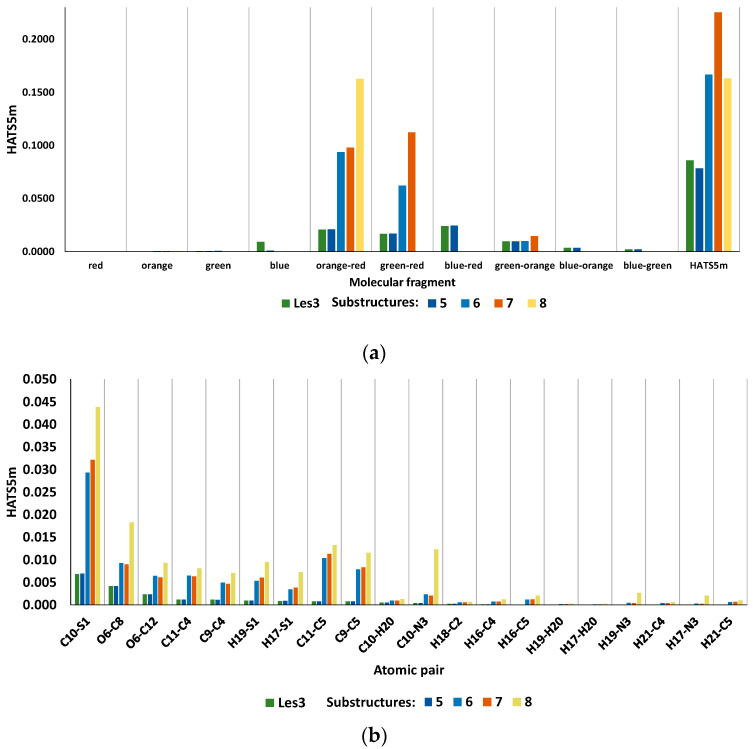
The contributions of Les3 (Les-3021) different fragments (**a**) and atomic pairs of orange-red (**b**) in the descriptor value. The numbering of atoms is shown in Figure 10.

A comparison of the inter-fragment effects of substructures 5, 6, and 7 reveals the impact of molecular size on leverages. Figure 12a and Table S4 show the contribution of different fragments of Les-3021 in the descriptor value. The increase in the descriptor value from substructure 5 to 6 is associated with contributions of atomic pairs in R1-Φ (orange-red) and R2-Φ (green-red) fragments due to leverage scores enhancement. Further downsizing molecule Les3 to substructure 7 leads to optimal leverage scores; thus, the highest HATS5m value. Atomic pairs contributions of the orange-red fragment are presented in Figure 12b, and for green-red fragment is shown in Figure S4. The contribution of R4 in the HATS5m value of Les3 mainly depends on the Cl43-C14 atomic pair (Figure S5). 

### 3.4. Impact of Molecular Shape

**Figure 13:**
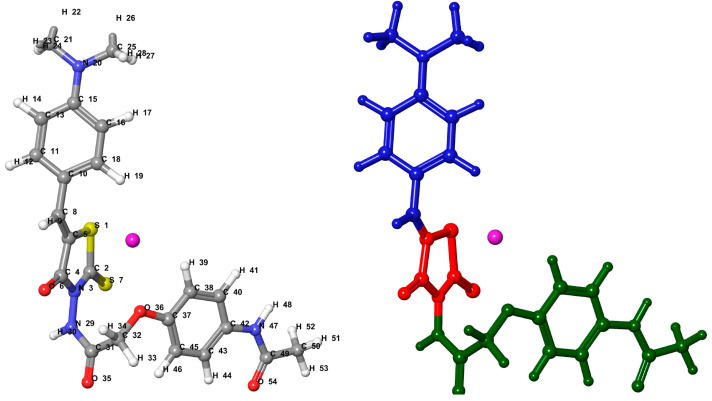
Les5 (Les-3834)—the numbering of atoms (left) and the split into fragments (common core Φ—red; R2—green; R4—blue). The geometric center of the molecule is marked pink.

Molecular similarity is a crucial topic in protein–ligand and protein–protein interaction recognition. Many molecular similarity evaluation approaches use molecular fingerprints, molecular alignment, pharmacophore, and 3D molecular descriptors. Combining HATS5m with a substructure-based approach and hierarchical cluster analysis leads to a view of the descriptor as a tool for differentiating the molecular structures according to their shape. A series of Les5 (Les-3834) conformers examine the similarity comparison. The numbering of atoms and the division Les5 into fragments are presented in Figure 13. 

**Figure 14:**
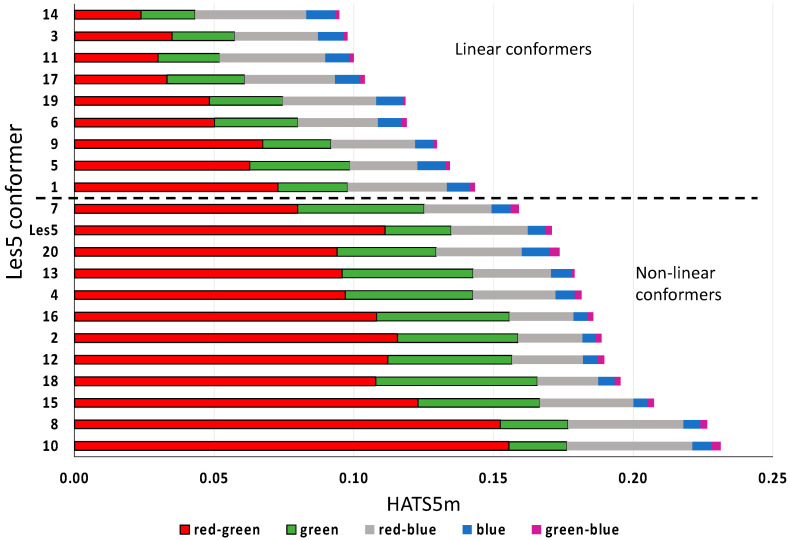
The contributions of 20 conformers of Les5 (Les-3834) to the HATS5m value. They are arranged in ascending order of the HATS5m value.

Twenty structures are generated using the Conformer Plugin of Chemaxon’s Marvin software. The method of Molecular mechanics (MM) is the same as in the publication [26] to maintain the comparability of the results. The Merck Molecular Force Field (MMFF94) is used with a normal optimization limit. The conformers are available in the Supplementary Materials (Structure-2.sdf in Supplementary Materials). The visual analysis of conformers indicates two groups relating to shape: nonlinear (conformers 2, 4, 7, 8, 10, 12, 13, 15, 16, 18, and 20 and molecule Les5) and linear (conformers 1, 3, 5, 6, 9, 11, 14, 17, and 19). Figure 14 shows the contributions of conformers arranged in ascending order of the HATS5m value. The nonlinear conformers have greater descriptor values than linear ones. In nonlinear conformers, the crucial factor is the mutual arrangement in space between the R2 substituent (green) and common core (red). In line with moving from nonlinear to linear conformers, a decrease in the descriptor value and an extension of contribution from other effects than those between green and red fragments is observed. For linear conformers, the essential contributions are derived from R2 and R4 and their inter-fragment effects regarding the common core (Figure 14). The arrangement of R2 and R4 to the Φ is well characterized by only seven and six atomic pairs, respectively (Figures S6 and S7). 

**Figure 15:**
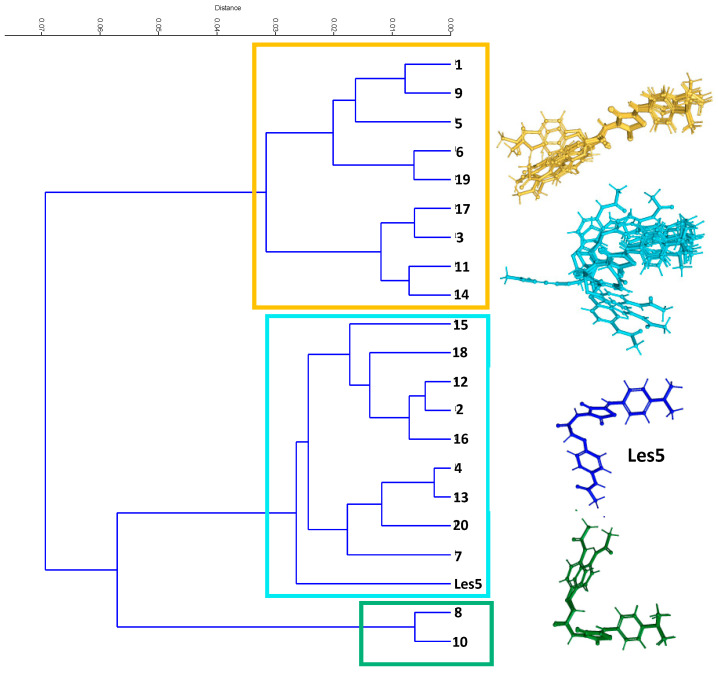
Results of cluster analysis based on atomic pairs contribution to the HATS5m value. The numbers denote the conformers of Les-5 molecule.

Furthermore, to investigate the conformers’ shape variability, cluster analysis was performed using the unweighted pair group method with arithmetic mean (UPGMA) and the Euclidean distance as a measure of similarity (Figure 15). The Cophenetic Correlation Coefficient (CCC) equal to 0.7659 validates the consistency of the hierarchical clustering pattern (dendrogram). Finally, the CCC measures the degree of preservation of the paired distances resulting from the dendrogram concerning the actual distances. The tree indicates the presence of three clusters in the data (Figure 15). The first cluster (yellow) corresponds to linear compounds from the original data set. The groups blue and green consist of nonlinear compounds. UPGMA demonstrates that two compounds (Les5_8, Les5_10) differ from the remaining nonlinear structures. Those two molecules exhibit an L-like shape compared to other nonlinear compounds, those with a U-like shape. The difference between the three-dimensional shapes of conformers is presented in Supplementary Materials (Video S2). 

The comparative analysis of contributions of atomic pairs enhances the interpretation of conformers. For further study, three compounds were selected: the original compound Les5, two conformers: 10 as nonlinear, and linear 14. The highest descriptor value had nonlinear conformer 10, intermediate Les5, while the lowest value had linear conformer 14. The Dirac-delta function indicated 117 atomic pairs distant at a topological distance of 5. However, each molecule had a different number of atomic pairs, which account for 80% of the HATS5m descriptor value, and their quantity is 21, 13, and 29 atomic pairs for Les5 and conformer 10 and 14, respectively.

Supplementary Video S3 shows an overview of the most valuable atomic pairs in compound Les5 and its conformers 10 and 14. The green-red fragment describes the atomic pairs determining the spatial arrangement of the 2-methoxyacetamide substructure of the R2 substituent. The blue-red fragment of these compounds consists mainly of atomic pairs derived from the common core and PhCH, where Ph = C_6_H_5_. The atomic pairs of the green substituent are essential to exploring the 3D geometry of the conformer 10, unlike the compound Les5. In conformer 14, the most critical atomic pairs describe the R4 substituent (blue) linear arrangement concerning the common core (red). The blue fragment is essential to characterize the linear shape of conformer 14, in contrast to Les5 and Les5_10.

### 3.5. Design of New 4-Thiazolidinone Derivatives

As shown above, the HATS5m descriptor is affected by many factors, including weighting scheme, molecular size, and shape. Therefore, to properly assess the intra- and inter-fragment contributions of individual substituents, the source compounds (Les) were modified (by the substructural method) to contain only the common core and the chosen substituent. The significance of substituents R1 (orange), R2 (green), and R4 (blue) was investigated with 46, 30, and 54 substructures, respectively. Finally, we prepared the group of 130 various substructures of 4-thiazolidinones derivatives. Molecular geometry re-optimization was not applied to keep the initial spatial arrangement of atomic pairs. All substructures are given in the Supplementary Materials (R1: Structure-3.sdf, R2: Structure-4.sdf, R4: Structure-5.sdf). The Supplementary Videos provide an overview of R1, R2, and R4 contributions to HATS5m (Videos S4–S6). The horizontal axis shows the contribution types (intra- and inter-fragment) and molecular descriptor HATS5m of each substructure. The vertical axis represents the magnitude of the contributions to the molecular descriptor. Additionally, the substructures are sorted in descending order of HATS5m value. In general, the inter-fragment contributions of substituents in HATS5m increase with decreasing molecular size and increasing number of sulfur, oxygen, nitrogen, chlorine, and bromine atoms located at the topological distance from another atom equal to five.

Based on the video files (Videos S4–S6), it is possible to select the substructures of 4-thiazolidinone derivatives with the favorable substituents providing the descriptor’s different values.

## 4. Materials and Methods

The set of 132 4-thiazolidinone derivatives, with biological activities taken from the literature, was used to develop an interpretation procedure of H-GETAWAY class of molecular descriptors on the example of HATS5m. In cooperation with the New Technologies Department of Softmaks.pl, the InterDescPy software tool was developed to explore the magnitudes of intra- and inter-fragment effects related to HATS5m. The InterDescPy was implemented in the Python programming language. In addition, the program provides the values of the atomic leverages and the contributions of the atomic pairs that compose the value of the HATS5m descriptor. Open-source PyMOL software was used to visualize the molecular structures leverage values of individual atoms. The geometry optimization of conformational sampling of Les5 (Les-3834) was performed by resorting to MarvinView 18.21.0 (2018), available from ChemAxon, by performing Molecular Mechanics calculations through the MMFF94 force field. The cluster analysis was carried out using a freeware statistical software PAST 4.03

## 5. Conclusions

This paper has attempted to highlight the need for interpretations of H-GETAWAY descriptors (focusing on the HATS5m) and has presented various factors that may affect the conclusions drawn. However, interpretation of the H-GETAWAY descriptors is complex and requires the simultaneous consideration of three components: weighting scheme, leverages, and the Dirac delta function. Each of these three components describes the molecular structure differently. The weighting scheme encodes the physicochemical property of atoms. Leverages encode the arrangement of atoms in three-dimensional space around the geometric center of the molecule. In turn, The Dirac-delta function topologically treats the 3D structure as a two-dimensional object. The HATS descriptor value is the sum of the contributions (expressed as a sum of the products of weights and leverages) of the atomic pairs indicated by the Dirac-delta function. The difficulty in interpreting is that the leverages are calculated for the whole molecule, not just for the atoms selected by the Dirac-delta function. Thus, the leverages of selected atomic pairs also depend on the arrangement of the other atoms in the molecule. The interpretation of H-GETAWAY requires adopting a substructure-based approach augmented with the cutting of molecules. 

## Data Availability

Not applicable.

## Supplementary Materials

The following are available online at https://www.mdpi.com/article/10.3390/ijms23126576/s1.
