# Supplementary material for: HATS5m as an Example of GETAWAY Molecular Descriptor in Assessing the Similarity/Diversity of the Structural Features of 4-Thiazolidinone
Source: Int J Mol Sci. 2022 Jun 12;23(12):6576. doi: 10.3390/ijms23126576 (PMC9223869; doi:10.3390/ijms23126576)
Supplement: Supplementary file 1 [file ijms-23-06576-s001.zip › Supplementary file.pdf]

Supplementary material

HATS5m as an example of GETAWAY molecular descriptor in assessing the similarity/diversity of the structural features of 4-thiazolidinone.

Mariusz Zapadka [1,*], Przemysław Dekowski [2] and Bogumiła Kupcewicz [1,*]

[1] Department of Inorganic and Analytical Chemistry, Faculty of Pharmacy, Nicolaus Copernicus University in Toruń, Jurasza 2, 85-089 Bydgoszcz, Poland
[2] New Technologies Department; Softmaks.pl Sp. z o.o., Kraszewskiego 1, 85-240 Bydgoszcz, Poland; przemyslaw.dekowski@softmaks.pl

* Correspondence: mariusz.zapadka@cm.umk.pl (M.Z.); kupcewicz@cm.umk.pl (B.K.),

Supplementary Figures

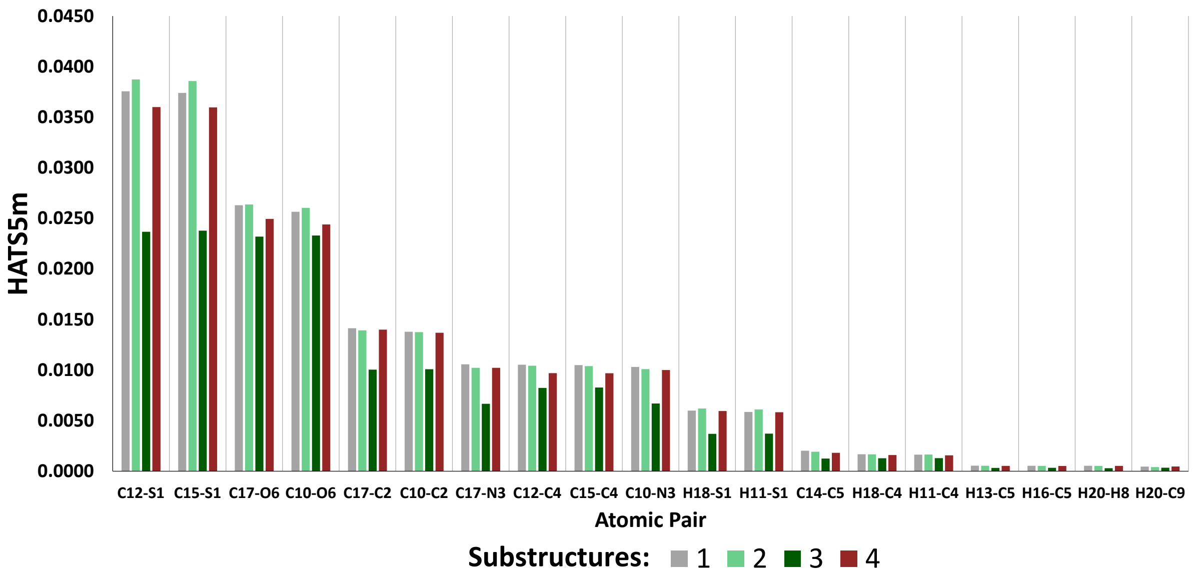

Figure S1. The impact of atomic pairs in red-blue (Φ-R4) fragments on HATS5m value.

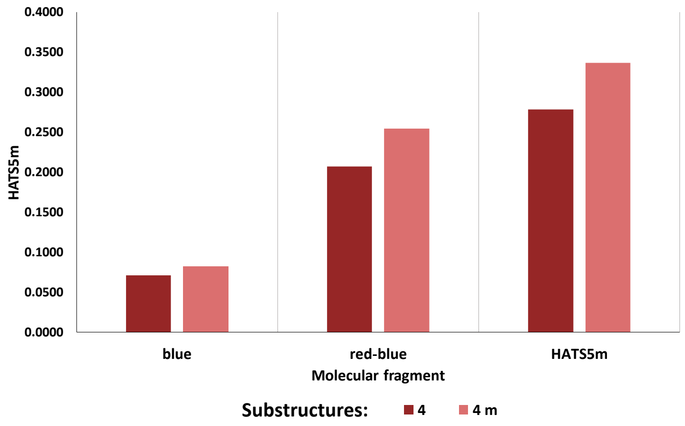

Figure S2. The impact of introducing a halogen atom in the ring at meta position on HAST5m descriptor value (4 – contained atom Br in the para position, 4 m – denoted of a molecule with Br in meta position).

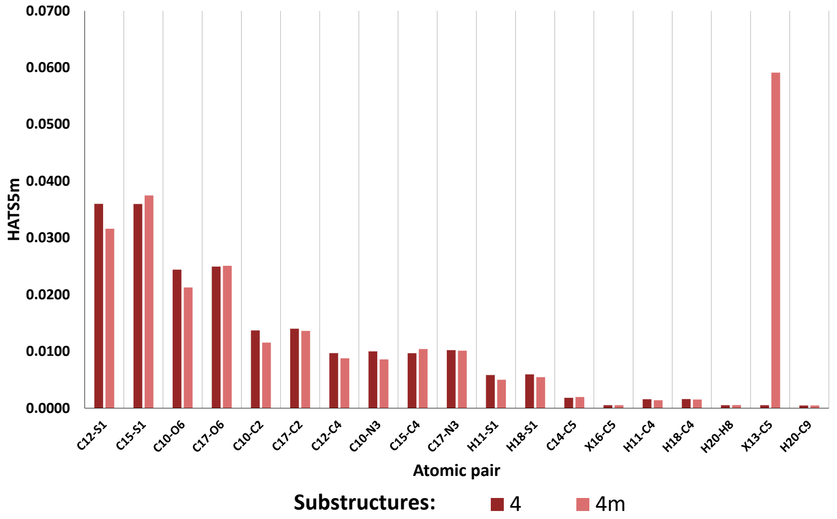

(**a**)

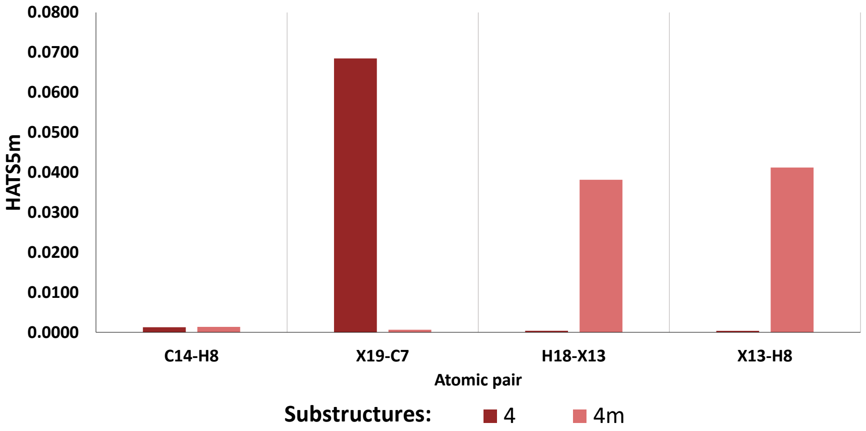

(**b**)

Figure S3. The contribution of atomic pairs included in: **(a)** blue-red fragments (R4-Φ); (**b**) the blue fragment (R4) of 4 and 4m substructures.

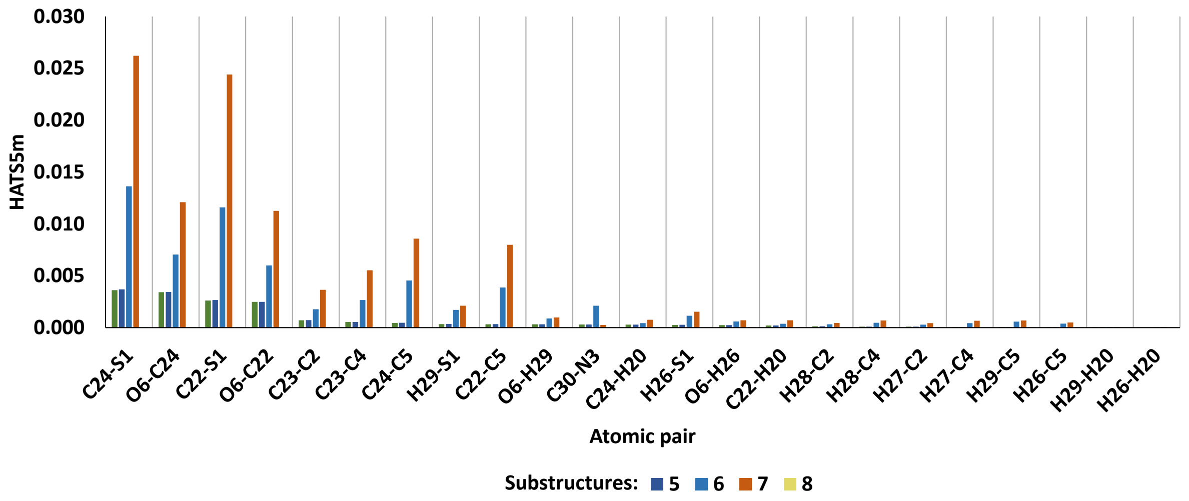

Figure S4. The contribution of atomic pairs included in green-red fragments (R2-Φ) of **Les3** (Les-3021) substructures.

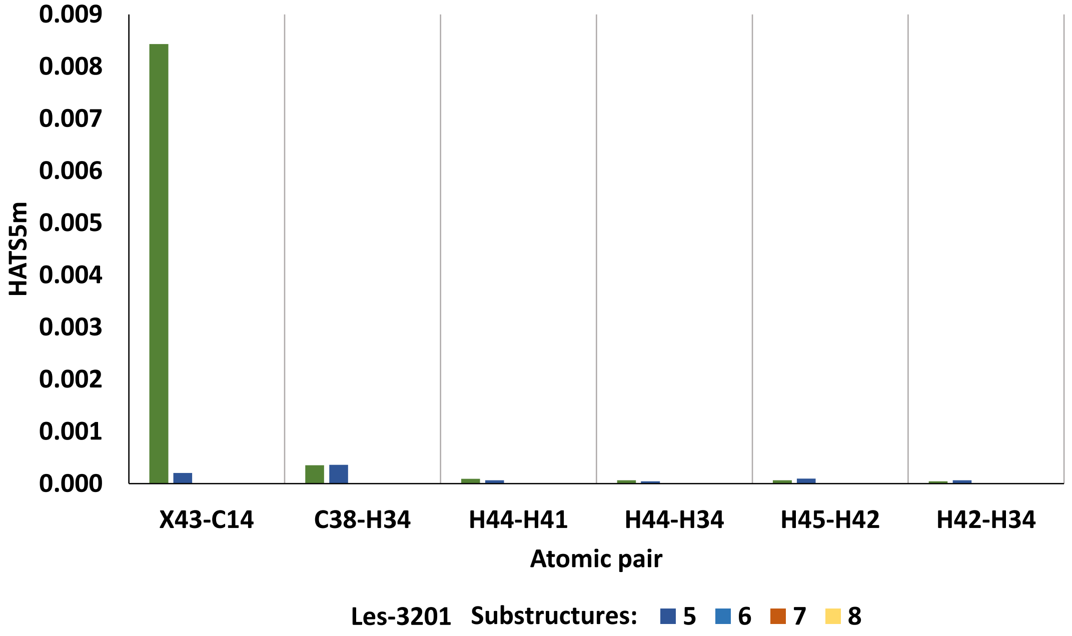

Figure S5. The contribution of R4 (blue) fragment in the HATS5m value of **Les3** (Les-3021) substructures.

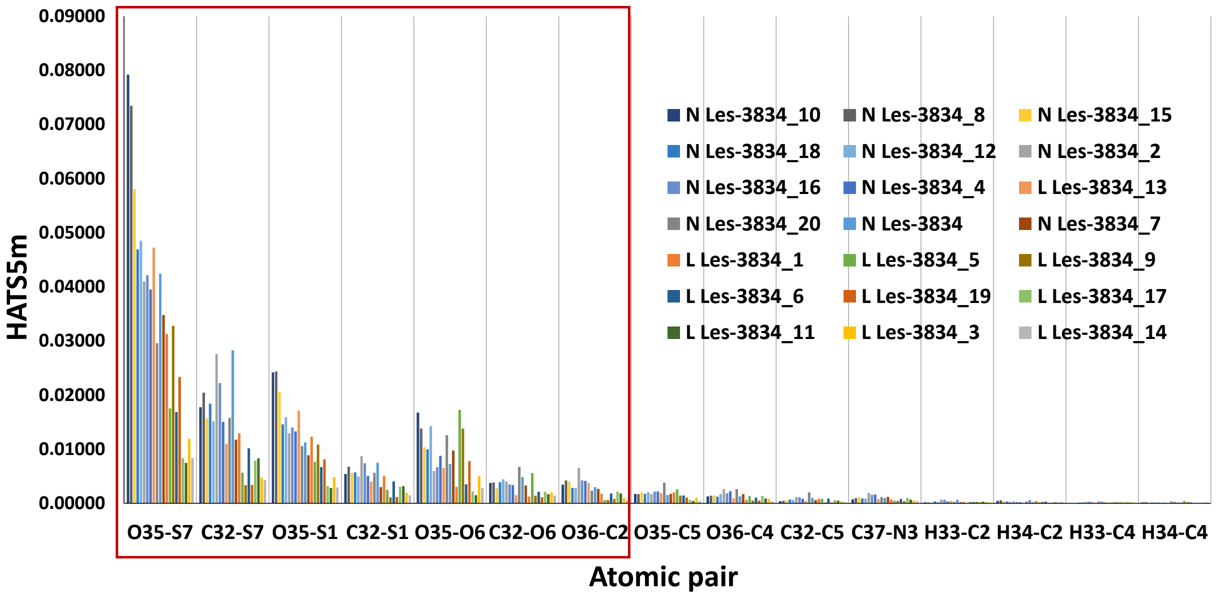

Figure S6. The contribution of atomic pairs included in green-red fragments (R2-Φ) of twenty conformers for **Les5** (Les-3834) molecule.

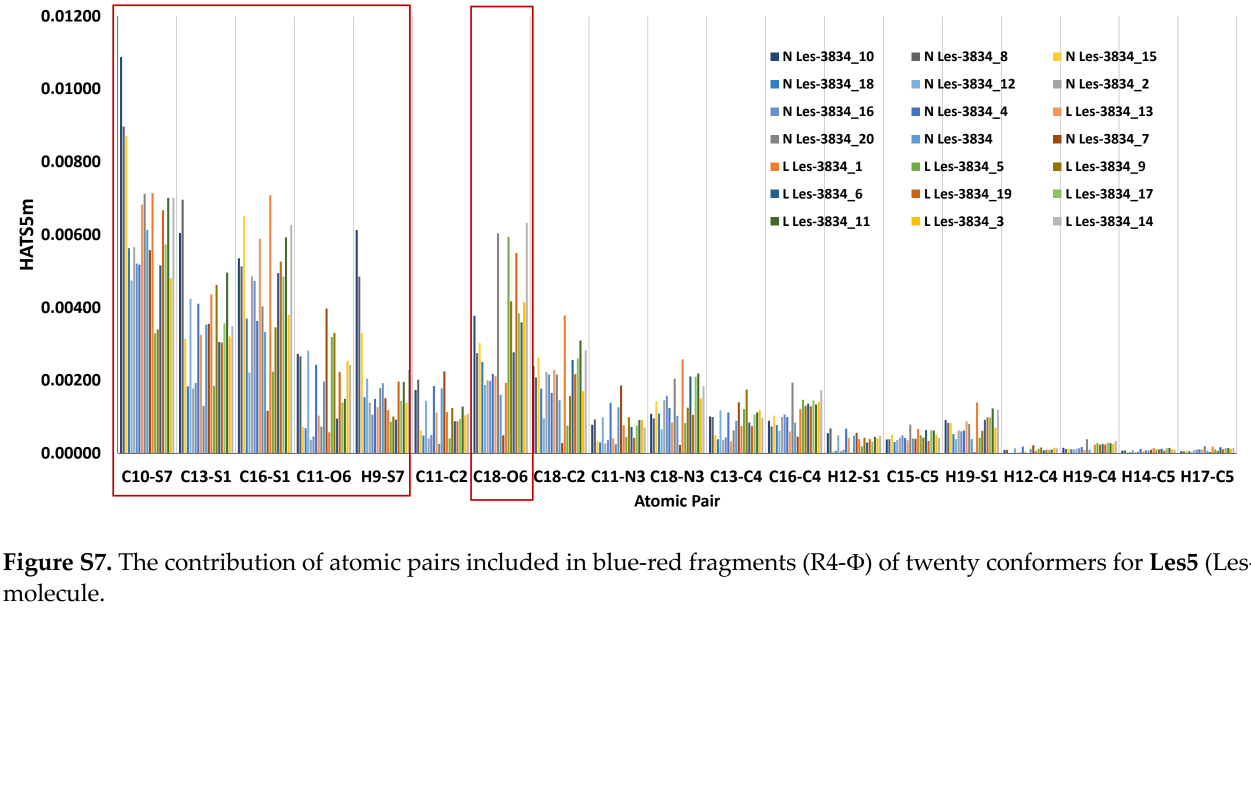

Figure S7. The contribution of atomic pairs included in blue-red fragments (R4-Φ) of twenty conformers for **Les5** (Les-3834) molecule.

Table S1. The contribution of fragments to HATS5m of fifteen Les molecules with the highest (1-15) and the lowest (16-30) molecular descriptor value.

No	Name	Group	red	red-orange	red-green	red-gray	red-blue	orange	orange-green	orange-gray	orange-blue	green	green-gray	green-blue	gray	gray-blue	blue	HATS5m
**1**	**Les-2283**	**2**	0	0	0.5937	0	0.0834	0	0	0	0	0.2381	0	0.0096	0	0	0.0095	0.9343
**2**	**Les-3731**	**2**	0	0	0.2577	0	0.1040	0	0	0	0	0.0248	0	0.0036	0	0	0.0577	0.4478
**3**	**Les-3748**	**2**	0	0	0.3472	0	0	0	0	0	0	0.0739	0	0	0	0	0	0.4211
**4**	**Les-4042**	**2**	0	0	0.3226	0	0.0772	0	0	0	0	0	0	0.0022	0	0	0.0082	0.4101
**5**	**Les-3783**	**2**	0	0	0.1039	0	0.1054	0	0	0	0	0.0612	0	0.0034	0	0	0.1218	0.3957
**6**	**Les-3849**	**2**	0	0	0.0352	0	0.1082	0	0	0	0	0.0519	0	0.0009	0	0	0.1917	0.3879
**7**	**Les-3424**	**1**	0	0.0340	0	0	0.0002	0.3169	0	0	0.0026	0	0	0	0	0	0	0.3537
**8**	**Les-3825**	**2**	0	0	0.1073	0	0.1148	0	0	0	0	0.0702	0	0.0118	0	0	0.0256	0.3296
**9**	**Les-3784**	**2**	0	0	0.1045	0	0.0840	0	0	0	0	0.0639	0	0.0034	0	0	0.0213	0.2771
**10**	**Les-2227**	**2**	0	0	0.1094	0	0.0604	0	0	0	0	0.0779	0	0.0014	0	0	0.0053	0.2543
**11**	**Les-4016**	**1**	0	0.1197	0.1197	0	0	0.0003	0.0137	0	0	0.0001	0	0	0	0	0.0000	0.2536
**12**	**Les-2028**	**3**	0	0	0	0.0804	0.1290	0	0	0	0	0	0	0	0.0228	0.0169	0.0000	0.2491
**13**	**Les-1029**	**1**	0	0.0392	0	0	0.0737	0.0105	0	0	0.0058	0	0	0	0	0	0.1154	0.2445
**14**	**Les-4032**	**2**	0	0	0.0851	0	0	0	0	0	0	0.1589	0	0	0	0	0	0.2439
**15**	**Les-772**	**3**	0	0	0	0.0665	0	0	0	0	0	0	0	0	0.1741	0	0	0.2407
**16**	**Les-3481**	1	0	0.001	0.0082	0	0.0070	0.0036	0.0012	0	0.0001	0.0003	0	0.001	0	0	0.0104	0.0329
**17**	**Les-4477**	2	0	0	0	0	0.0075	0	0	0	0	0	0	0.001	0	0	0.0327	0.0414
**18**	**Les-4376**	2	0	0	0.0154	0	0	0	0	0	0	0.029	0	0.001	0	0	0	0.0459
**19**	**Les-316**	1	0	0.001	0.0150	0	0.0119	0.0056	0.0019	0	0.0003	0.0004	0	0.002	0	0	0.0078	0.0465
**20**	**Les-4580**	1	0	0	0	0	0.0249	0	0	0	0	0	0	0	0	0	0.0223	0.0473
**21**	**Les-4604**	1	0	0.002	0	0	0.0001	0.0469	0	0	0.0010	0	0	0	0	0	0	0.0504
**22**	**Les-4579**	1	0	0.023	0	0	0.0001	0.0238	0	0	0.0062	0	0	0	0	0	0	0.0533
**23**	**Les-4577**	1	0	0.005	0	0	0.0000	0.0458	0	0	0.0027	0	0	0	0	0	0	0.0537
**24**	**Les-3701**	1	0	0.027	0	0	0	0.0249	0	0	0.0028	0	0	0	0	0	0	0.0546
**25**	**Les-4606**	1	0	0.020	0	0	0.0072	0.0229	0	0	0.0073	0	0	0	0	0	0	0.0572
**26**	**Les-3882**	1	0	0.005	0	0	0.0176	0.0217	0	0	0.0008	0	0	0	0	0	0.0120	0.0573
**27**	**Les-3497**	1	0	0.003	0.0215	0	0.0163	0.0053	0.0034	0	0.0006	0.001	0	0.003	0	0	0.0035	0.0574
**28**	**Les-2379**	1	0	0.006	0	0	0.0178	0.0049	0	0	0.0010	0	0	0	0	0	0.0279	0.0575
**29**	**Les-4599**	1	0	0.005	0	0	0.0001	0.0498	0	0	0.0028	0	0	0	0	0	0	0.0580
**30**	**Les-4601**	1	0	0.007	0	0	0	0.0515	0	0	0	0	0	0	0	0	0	0.0581

Table S2. The influence of the atomic mass on the descriptor value presented as the contribution of intra- and inter-fragments effect in the HATS5m value.

Name	red	blue	red-blue	HATS5m
1	0.0000	0.0036	0.2161	0.2196
2	0.0000	0.0156	0.2181	0.2337
3	0.0000	0.0287	0.1576	0.1862
4	0.0000	0.0713	0.2074	0.2787

Table S3. The contributions (%) of atomic pairs to **HATS5(w)** value depending on the weighting scheme (w). Atomic pairs are ordered according to decreasing contribution expressed as % of HATS5(w). Colors indicate atomic pairs covered approximately 80% of descriptor value. Descriptor values are shown in the last row of the table.

Unweighted		Weight: atomic mass		Weight: vdV volume		Weight: electronegativity		Weight: polarizability		Weight: ionization potential	
Atomic pair	% of HATS5u	Atomic pair	% of HATS5m	Atomic pair	% of HATS5v	Atomic pair	% of HATS5e	Atomic pair	%HATS5p	Atomic pair	%HATS5i
**H20-H8**	**13.61**	**Br19-C7**	**24.59**	**C12-S1**	**7.84**	**H20-H8**	**12.45**	**C12-S1**	**9.26**	**H20-H8**	**15.33**
**H13-H18**	**9.73**	**C12-S1**	**12.92**	**C15-S1**	**7.84**	**H13-H18**	**8.90**	**C15-S1**	**9.25**	**H13-H18**	**10.96**
**H11-H16**	**9.49**	**C15-S1**	**12.91**	**Br19-C7**	**7.62**	**H11-H16**	**8.68**	**Br19-C7**	**7.44**	**H11-H16**	**10.68**
**H13-H8**	**9.48**	**C17-O6**	**8.95**	**C17-C2**	**7.48**	**H13-H8**	**8.68**	**H18-S1**	**6.94**	**H13-H8**	**10.68**
**H16-H8**	**9.43**	**C10-O6**	**8.75**	**C10-C2**	**7.32**	**H16-H8**	**8.63**	**H11-S1**	**6.81**	**H16-H8**	**10.62**
**H18-S1**	**4.85**	**C17-C2**	**5.02**	**C12-C4**	**5.18**	**H18-S1**	**5.07**	**C17-C2**	**5.83**	**H18-S1**	**4.16**
**H11-S1**	**4.76**	**C10-C2**	**4.91**	**C15-C4**	**5.18**	**H11-S1**	**4.97**	**C10-C2**	**5.71**	**H11-S1**	**4.08**
**H18-C4**	**3.49**	**C17-N3**	**3.67**	**C17-O6**	**5.13**	**C17-O6**	**4.69**	**H20-H8**	**4.50**	**H18-C4**	**3.25**
**C17-O6**	**3.43**	C10-N3	3.59	**C10-O6**	**5.01**	**C10-O6**	**4.58**	**C12-C4**	**4.04**	**C17-O6**	**3.20**
**H11-C4**	**3.42**	C12-C4	3.48	**H18-S1**	**4.61**	**H18-C4**	**3.39**	**C15-C4**	**4.04**	**H11-C4**	**3.19**
**C10-O6**	**3.35**	C15-C4	3.48	**H11-S1**	**4.53**	**H11-C4**	**3.32**	**C17-O6**	**3.55**	**C10-O6**	**3.13**
**C14-H8**	**2.76**	H18-S1	2.14	**H20-H8**	**3.56**	**C12-S1**	**2.74**	**C10-O6**	**3.47**	**C14-H8**	**2.58**
**C17-C2**	**2.56**	H11-S1	2.09	**C17-N3**	**3.26**	**C15-S1**	**2.73**	**H13-H18**	**3.22**	C17-C2	1.98
C10-C2	2.50	C14-C5	0.65	**C10-N3**	**3.18**	**C14-H8**	**2.68**	**H11-H16**	**3.14**	C10-C2	1.93
C12-S1	2.47	H18-C4	0.58	**H18-C4**	**3.05**	C17-C2	2.64	**H13-H8**	**3.14**	C12-S1	1.75
C15-S1	2.46	H11-C4	0.56	H11-C4	2.99	C10-C2	2.58	H16-H8	3.12	C15-S1	1.75
Br19-C7	1.88	C14-H8	0.46	H13-H18	2.54	Br19-C7	2.27	H18-C4	3.03	C17-N3	1.60
C12-C4	1.77	H20-H8	0.19	H11-H16	2.48	C17-N3	1.92	H11-C4	2.97	C10-N3	1.56
C15-C4	1.77	H13-C5	0.19	H13-H8	2.48	C10-N3	1.87	C14-H8	2.40	Br19-C7	1.52
C17-N3	1.60	H16-C5	0.19	H16-H8	2.46	C12-C4	1.83	C17-N3	2.28	C12-C4	1.37
C10-N3	1.57	H20-C9	0.17	C14-H8	2.41	C15-C4	1.83	C10-N3	2.23	C15-C4	1.37
H13-C5	1.14	H13-H18	0.13	H13-C5	1.00	H13-C5	1.11	H13-C5	0.99	H13-C5	1.06
H16-C5	1.13	H11-H16	0.13	H16-C5	0.99	H16-C5	1.10	H16-C5	0.98	H16-C5	1.06
H20-C9	1.01	H13-H8	0.13	C14-C5	0.97	H20-C9	0.98	H20-C9	0.88	H20-C9	0.94
C14-C5	0.33	H16-H8	0.13	H20-C9	0.88	C14-C5	0.34	C14-C5	0.76	C14-C5	0.26
**0.5468**		**0.2787**		**0.1870**		**0.3502**		**0.2399**		**0.7085**	

Table S4. The influence of molecular size on the descriptor value presented as the contribution of intra- and inter- fragments effects in the HATS5m value of **Les3** (Les-2301) molecule.

Name	red	orange	green	blue	orange-red	green-red	blue-red	green-orange	blue-orange	blue-green	HATS5m
**Les1**	0.000	0.0002	0.0005	0.0091	0.0208	0.0166	0.0239	0.0095	0.0033	0.0020	**0.0859**
**5**	0.000	0.0002	0.0005	0.0008	0.0209	0.0168	0.0244	0.0095	0.0034	0.0020	**0.0785**
**6**	0.000	0.0003	0.0007	0.0000	0.0937	0.0622	0.0000	0.0098	0.0000	0.0000	**0.1667**
**7**	0.000	0.0003	0.0001	0.0000	0.0980	0.1123	0.0000	0.0145	0.0000	0.0000	**0.2253**
**8**	0.000	0.0004	0.0000	0.0000	0.1627	0.0000	0.0000	0.0000	0.0000	0.0000	**0.1631**
